# Supplementary material for: Phylodynamic of SARS-CoV-2 during the second wave of COVID-19 in Peru
Source: Nat Commun. 2023 Jun 15;14:3557. doi: 10.1038/s41467-023-39216-8 (PMC10272135; doi:10.1038/s41467-023-39216-8)
Supplement: Supplementary file 1 — Supplementary Information [file 41467_2023_39216_MOESM1_ESM.pdf]

Phylodynamic of SARS-CoV-2 during the second wave of COVID-19 in Peru

Santiago Justo Arevalo*[,1,2,3], Carmen Sofia Uribe Calampa[1], Cinthy Jimenez Silva[4], Mauro Quiñones Aguilar[1], Remco Bouckaert[5], Joao Renato Rebello Pinho[2,6]

1.- Facultad de Ciencias Biológicas, Universidad Ricardo Palma, Lima – Peru.

2.- Laboratório Clínico do Hospital Israelita Albert Einstein, São Paulo – Brasil

3.- Departamento de Bioquímica, Instituto de Química, Universidade de São Paulo, São Paulo – Brasil

4.- School of Biological Sciences, University of Auckland, Auckland – New Zealand

5.- School of Computer Science, University of Auckland, Auckland, – New Zealand

6.- Department of Gastroenterology and Pathology, University of São Paulo School of Medicine, São Paulo, Brazil

*Correspondence to: Santiago Justo Arevalo, santiago.justo@urp.edu.pe

Supporting Information

Contents:

Supplementary Figures

Figure S1. Number of Lambda genomes by week in Peru and the rest of the world.

Figure S2. Relative prevalence of Lambda genomes in six different countries.

Figure S3. Number of genomes and Lambda genomes in six different countries.

Figure S4. Number of total genomes and Lambda genomes in six different countries and sampling proportion by country.

Figure S5. Correlation between number of Lambda genomes and estimated Lambda cases of the full dataset (before sampling) and after the sampling considering estimated Lambda cases between January, 2021, and September, 2021, in each country.

Figure S6. R values of the correlation between root-to-tip distance and sequence sampling time, and informative quartets of samples of Lambda genomes considering different intervals of time.

Figure S7. Distribution of substitution rates of Lambda in different samples.

Figure S8. Sampling of Lambda genomes from three different countries based on the number of cases to improve correlation between number of genomes and number of cases.

Figure S9. Sampling of Lambda genomes from three different countries based on the number of cases to improve correlation between number of genomes and number of cases adding genomes from initial weeks of Lambda waves.

Figure S10. Estimations of date and location of the origin of Lambda from samples considering genomes from the early weeks of Lambda waves in each country.

Figure S11. Characteristic mutations of Lambda sublineages.

Figure S12. Characteristic mutations of Gamma sublineages.

Figure S13. Correlation between number of SubL1+L2 genomes and estimated SubL1+L2 cases of the full dataset (before sampling) and after the sampling considering estimated SubL1+L2 cases in each Peruvian region.

Figure S14. Correlation between number of Gamma sublineages genomes and estimated Gamma sublineages cases of the full dataset (before sampling) and after the sampling considering estimated Gamma sublineages cases in each Peruvian region.

Figure S15. Estimations of location of the origin of Gamma and Lambda sublineages in different samples.

Figure S16. Number of transitions of sublineages between Peruvian regions in differen samples.

Supplementary Tables

Table S1. Classification of Peruvian cities in regions.

Table S2. Temporal Signal of each sublineage after sampling considering estimated cases in each Peruvian region.

Table S3. Distribution of quartets of each sublineage after sampling considering estimated cases in each Peruvian region.

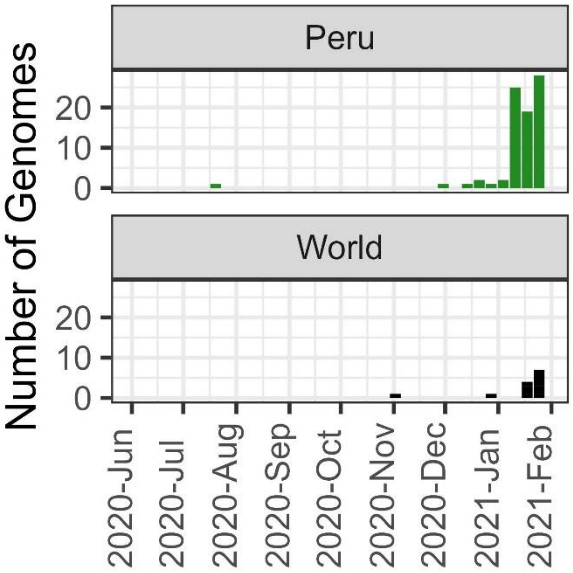

Figure S1. Number of Lambda genomes by week in Peru and the rest of the world. Above) Number of Lambda genomes by week of collection in Peru. Below) Number of Lambda genomes by collection week in countries different to Peru.

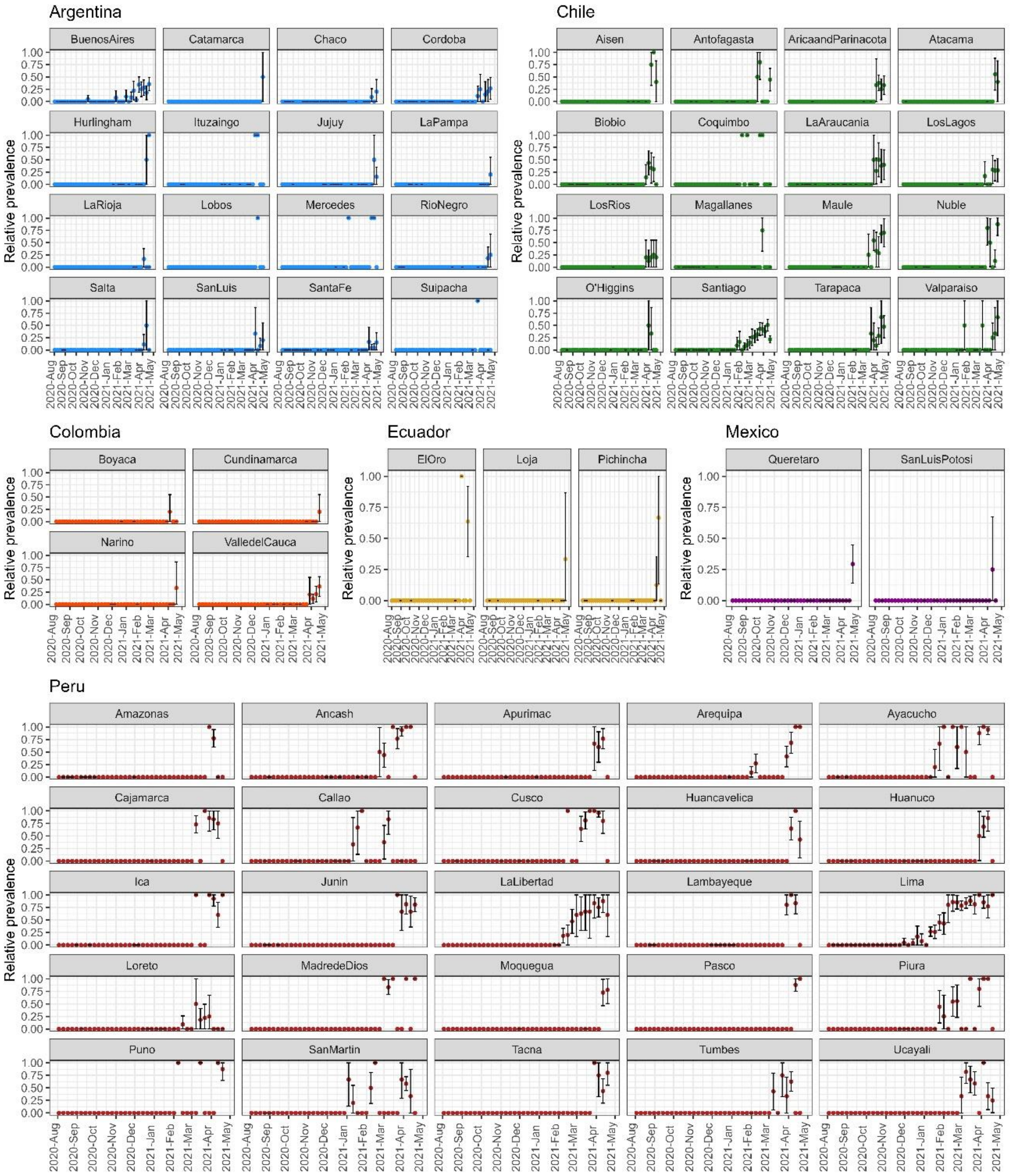

Figure S2. Relative prevalence of Lambda genomes in six different countries. Relative prevalence by week of Lambda in each city from countries with at least two cities that had a Lambda prevalence higher than 15 % before April 2021. Error bars represent the 95 % confidence interval of the estimate.

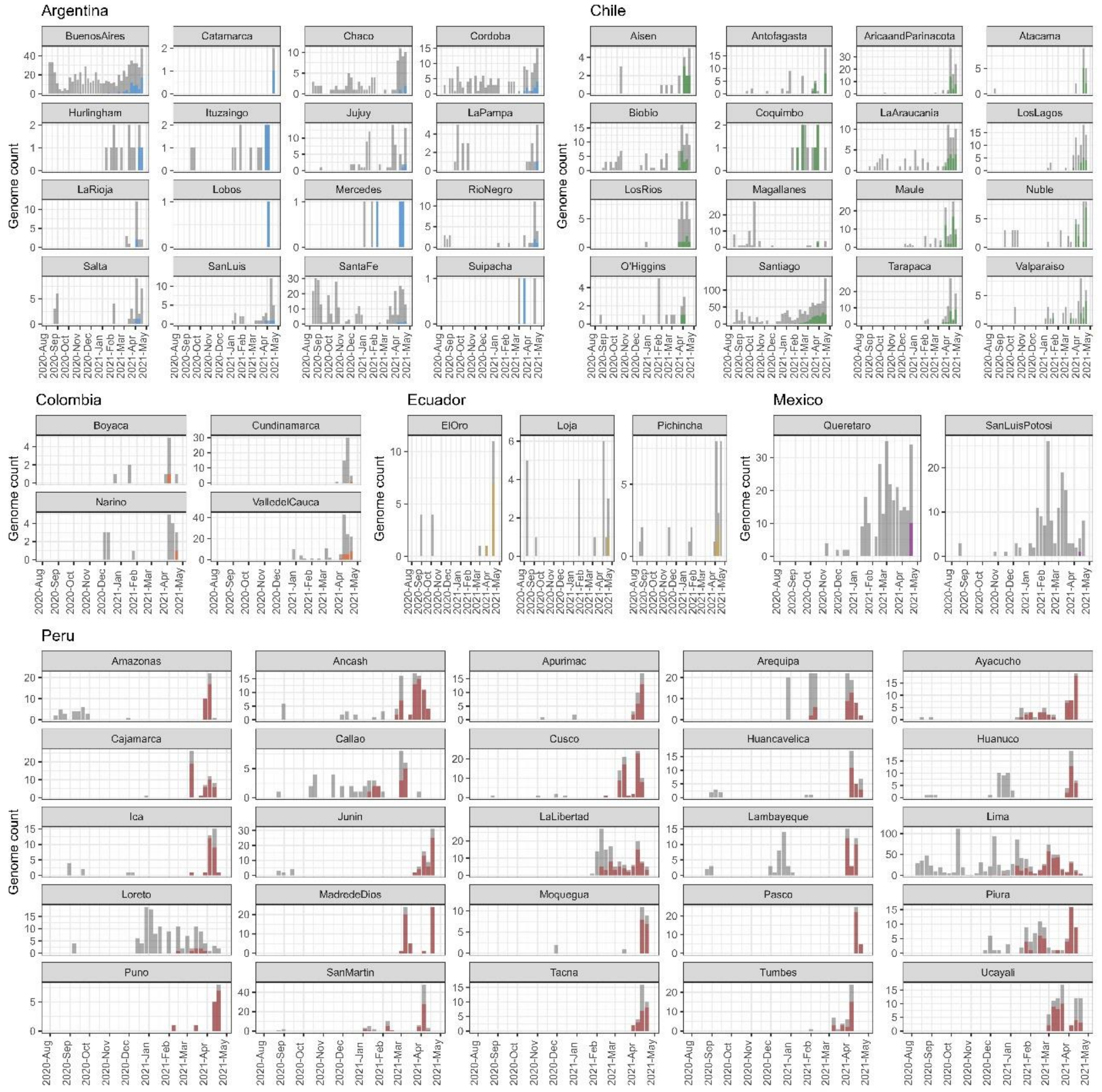

Figure S3. Number of genomes and Lambda genomes in six different countries. Number of genomes (grey bars) and Lambda genomes (colored bars) by week in each city with from countries that had at least two cities that had a Lambda prevalence higher than 15 % before April 2021. Error bars represent the 95 % confidence interval of the estimate.

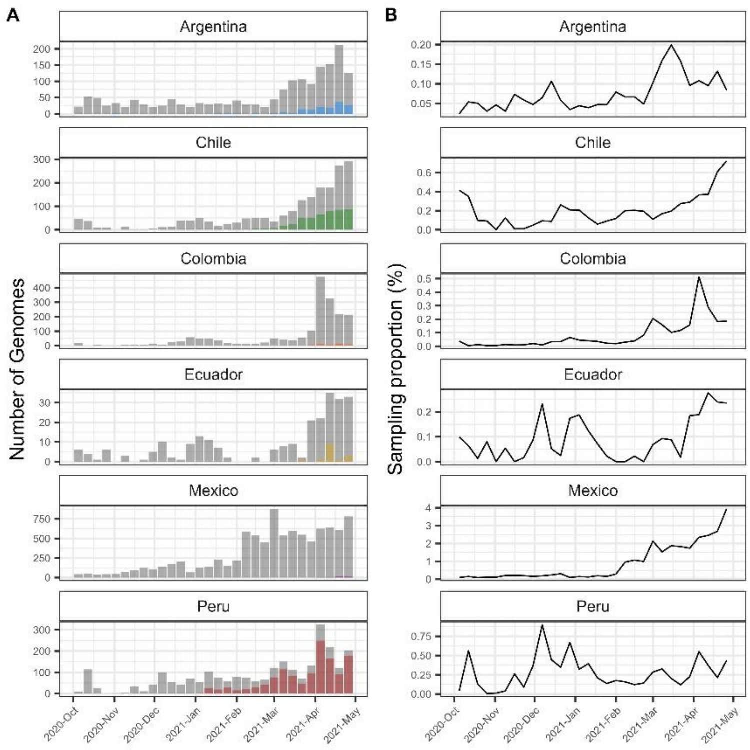

Figure S4. Number of genomes and Lambda genomes in six different countries and sampling proportion by country. A) Number of genomes (grey bars) and Lambda genomes (colored bars) by week in each country with at least two cities that had a Lambda prevalence higher than 15 % before April 2021. B) Sampling proportion (number of sequenced genomes divided by number of reported cases) by week in each analysed country.

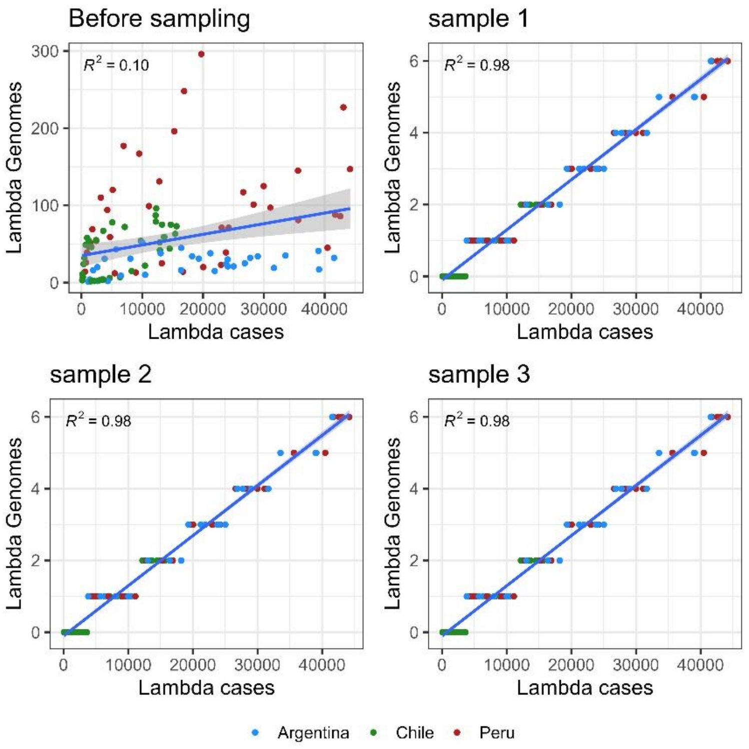

Figure S5. Correlation between number of Lambda genomes and estimated Lambda cases of the full dataset (before sampling) and after the sampling considering estimated Lambda cases between January, 2021, and September, 2021, in each country. Each point represents the number of Lambda genomes and the estimated Lambda cases in a specific country-week combination. Points are coloured according to the country. Grey ribbons represent the 95 % confidence interval of the regression line. R and p values of pearson correlation are shown in each panel. n = 75.

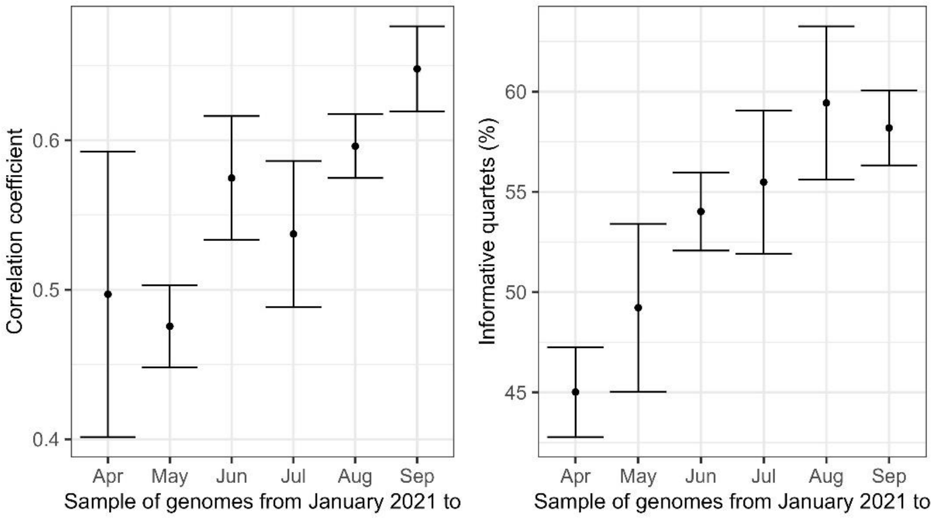

Figure S6. Correlation between root-to-tip distance and sequence sampling time, and informative quartets of samples of Lambda genomes considering different intervals of time. Lambda genomes were sampled considering the estimated number of Lambda genomes and considering different intervals of time from January, 2021 to different months (x-axis). A maximum likelihood phylogeny was reconstructed using these samples and the correlation coefficient (R value) was calculated using Tempest. Informative quartets were obtained by likelihood mapping of the samples using IQTREE2. Points indicate the mean and error bars shown the standard deviation of three different samples (n = 3).

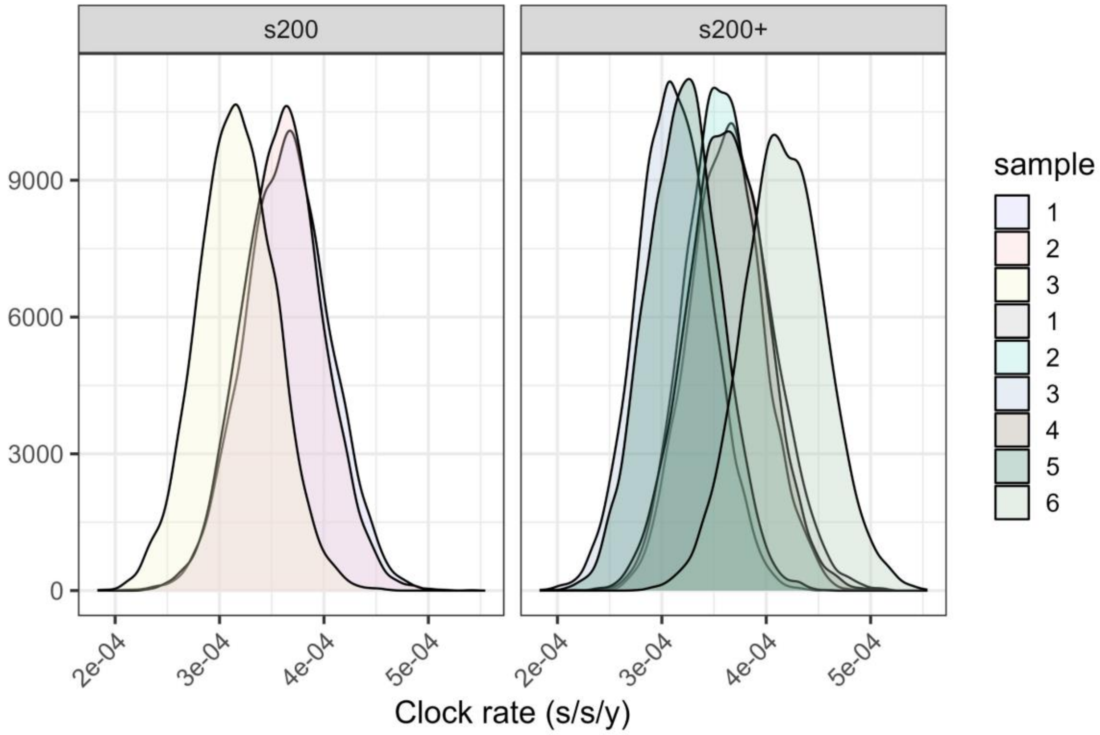

Figure S7. Distribution of substitution rates of Lambda in different samples. Substitution rates are expressed in substitution per site per year (s/s/y). s200 means the samples without addition of genomes from early weeks and S200+ means the samples with addition of genomes from early weeks. The number of trees (n) from the posterior distributions that were analysed were 391, 349, and 366 for samples 1, 2, and 3, of s200, respectively, and 1077, 973, 1041, 965, 1045, and 954 for samples 1, 2, 3, 4, 5. and 6, of s200+, respectively.

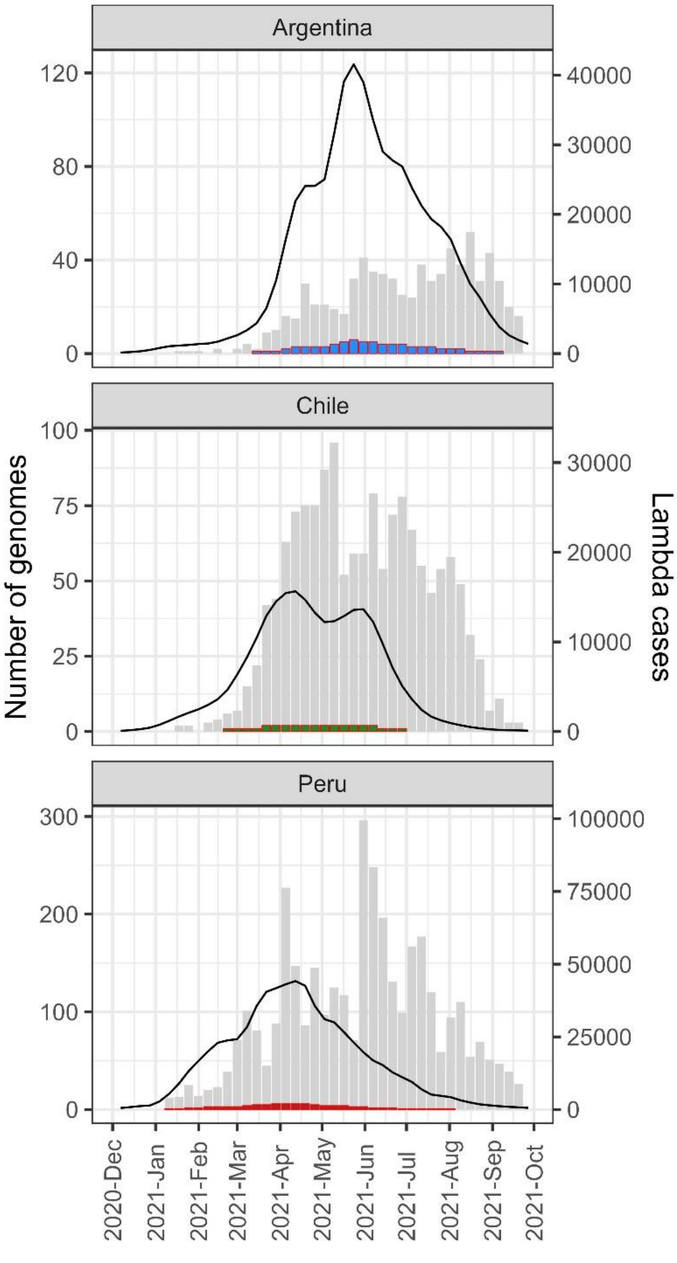

Figure S8. Sampling of Lambda genomes from three different countries based on the number of cases to improve correlation between number of genomes and number of cases. Black line represents the number of estimated Lambda cases, grey bars represent the total number of available Lambda genomes, coloured bars represent the number of genomes present in the sample to be analysed, and bars with red borderlines represent the ideal number of genomes to be picked by week to obtain a perfect correlation between number of genomes and number of cases.

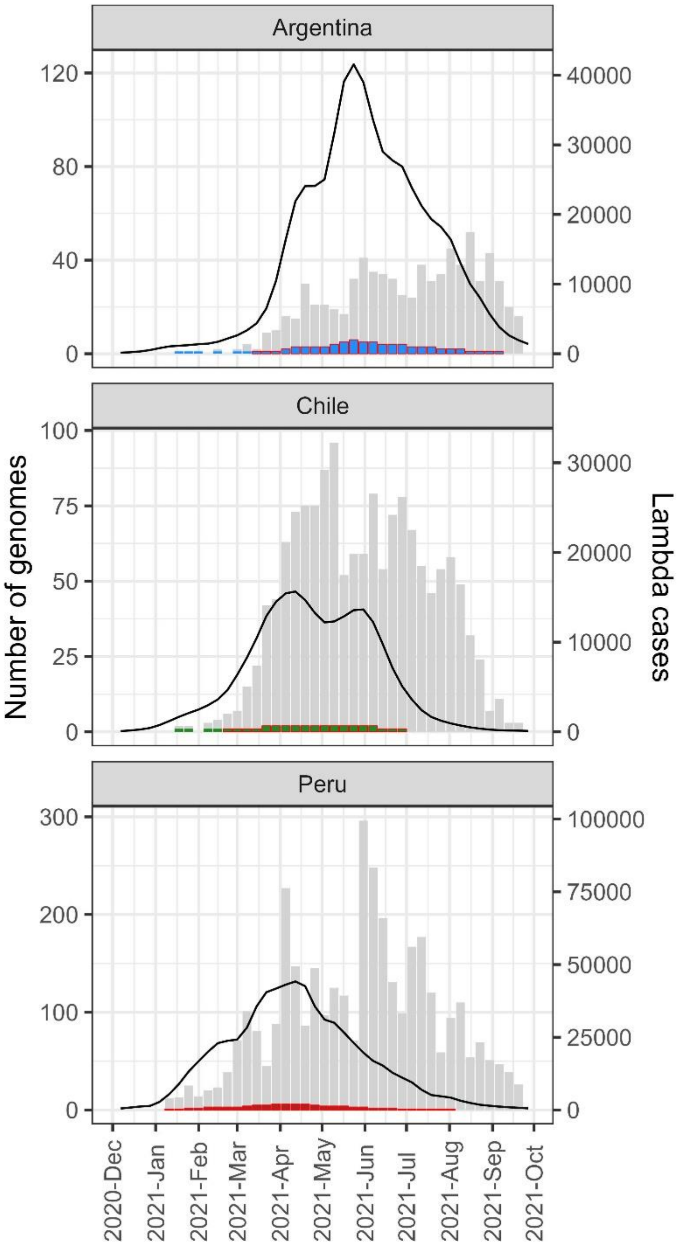

Figure S9. Sampling of Lambda genomes from three different countries based on the number of cases to improve correlation between number of genomes and number of cases adding genomes from initial weeks of Lambda waves. Black line represents the number of estimated Lambda cases, grey bars represent the total number of available Lambda genomes, coloured bars represent the number of genomes present in the sample to be analysed, and bars with red borderlines represent the ideal number of genomes to be picked by week to obtain a perfect correlation between number of genomes and number of cases.

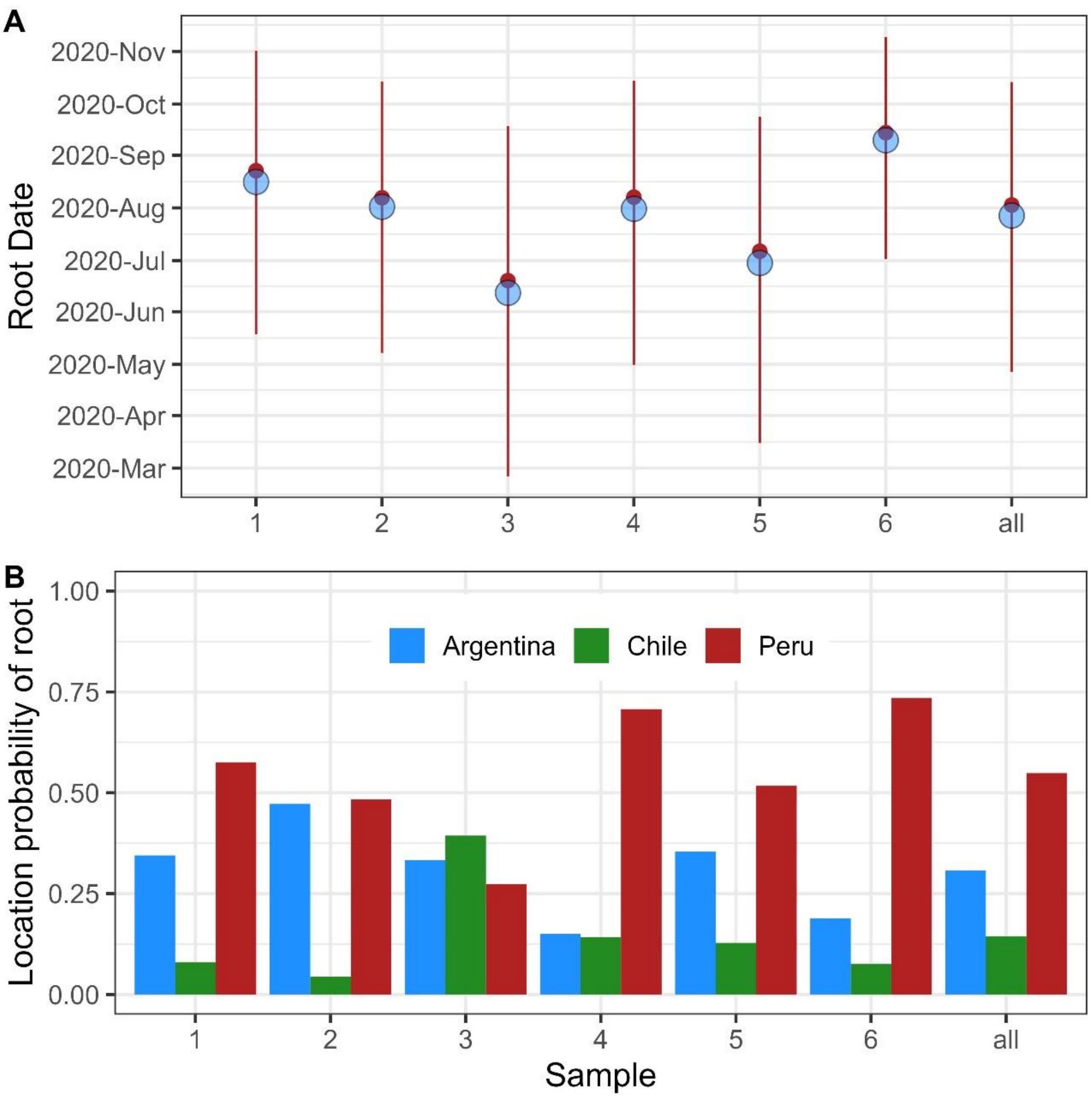

Figure S10. Estimations of date and location of the origin of Lambda from samples considering genomes from the early weeks of Lambda waves in each country. A) High posterior density 95 % of the root date that represent the time of origin of the lineage Lambda. Blue circles and red points represent the mean and the median, respectively. B) Location probability of the root that represents the MRCA of the Lambda lineage. In A and B, the results of three analysis with different samples are shown (1 – 6) together with the combined results of these six samples (all). The number of trees (n) from the posterior distributions that were analysed were 1077, 973, 1041, 965, 1045, and 954 for samples 1, 2, 3, 4, 5 and 6, respectively.

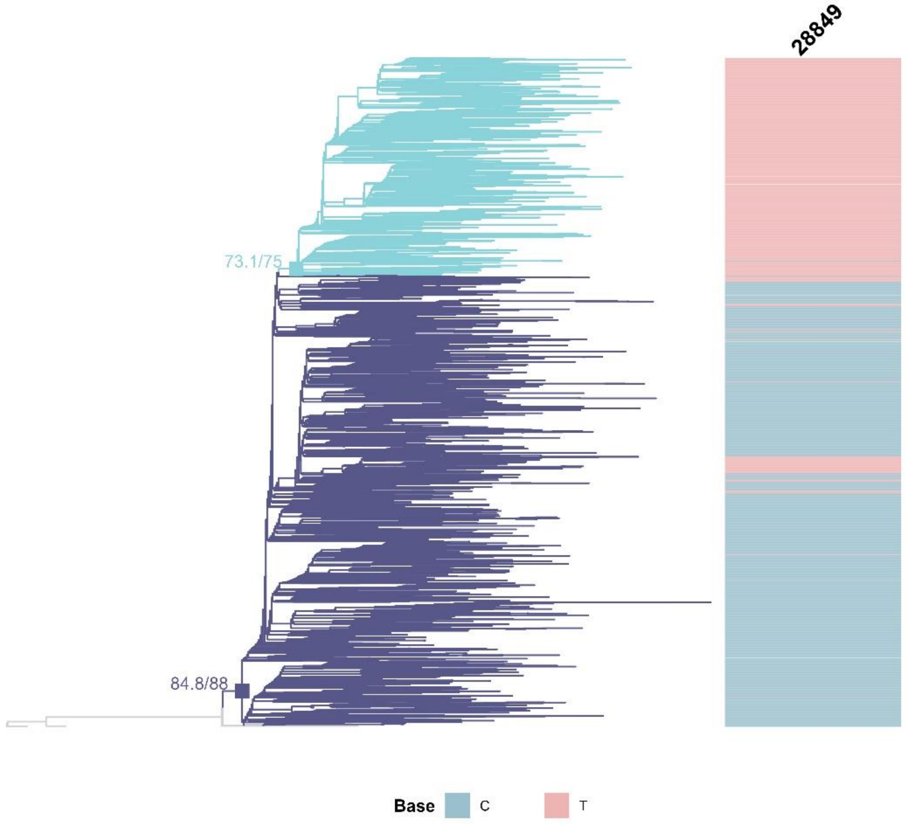

Figure S11. Characteristic mutations of Lambda sublineages. Maximum likelihood trees of Lambda genomes (n = 3461) from Peru showing two Lambda sublineages: purple (SubL1) and cyan (SubL2). The nodes are labelled by its support (SH-aLRT/bootstrap). At the right, it is shown the nucleotide position 28849 coloring according to its state (C or T in blue or pink, respectively).

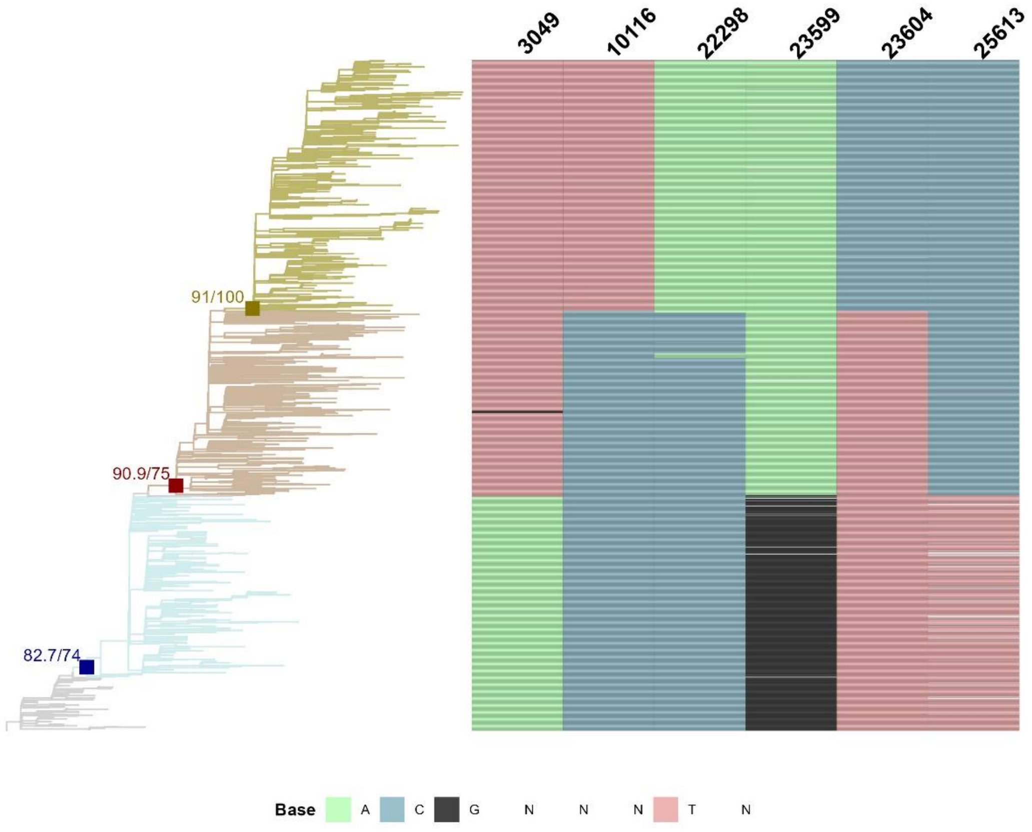

Figure S12. Characteristic mutations of Gamma sublineages. Maximum likelihood trees of Gamma genomes (n = 1674) from Peru showing three Gamma sublineages: cyan (SubG1), brown (SubG2), and yellow (SubG3). The nodes are labelled by its support (SH-aLRT/bootstrap). At the right, it is shown the nucleotide positions 3049, 10116, 22298, 23599, 23604, 25613 coloring according to its state: A in green, C in blue, G in black, and T in pink.

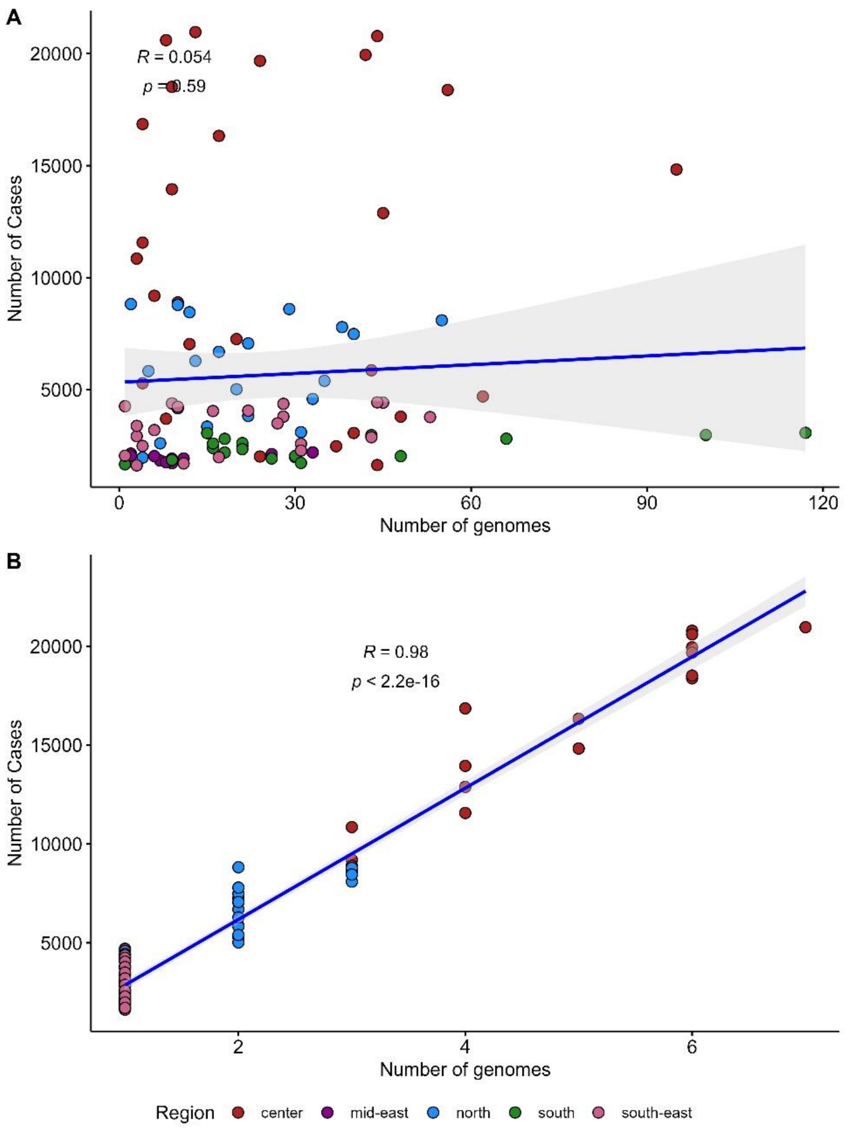

Figure S13. Correlation between number of SubL1+L2 genomes and estimated SubL1+L2 cases of the full dataset (before sampling (A)) and after the sampling (B) considering estimated SubL1+L2 cases in each Peruvian region. Each point represents the number of SubL1+L2 genomes and the estimated SubL1+L2 cases in a specific region-week combination. Points are coloured according to the region. Grey ribbons represent the 95 % confidence interval of the regression line. R and p values of pearson correlation are shown in each panel. n = 102.

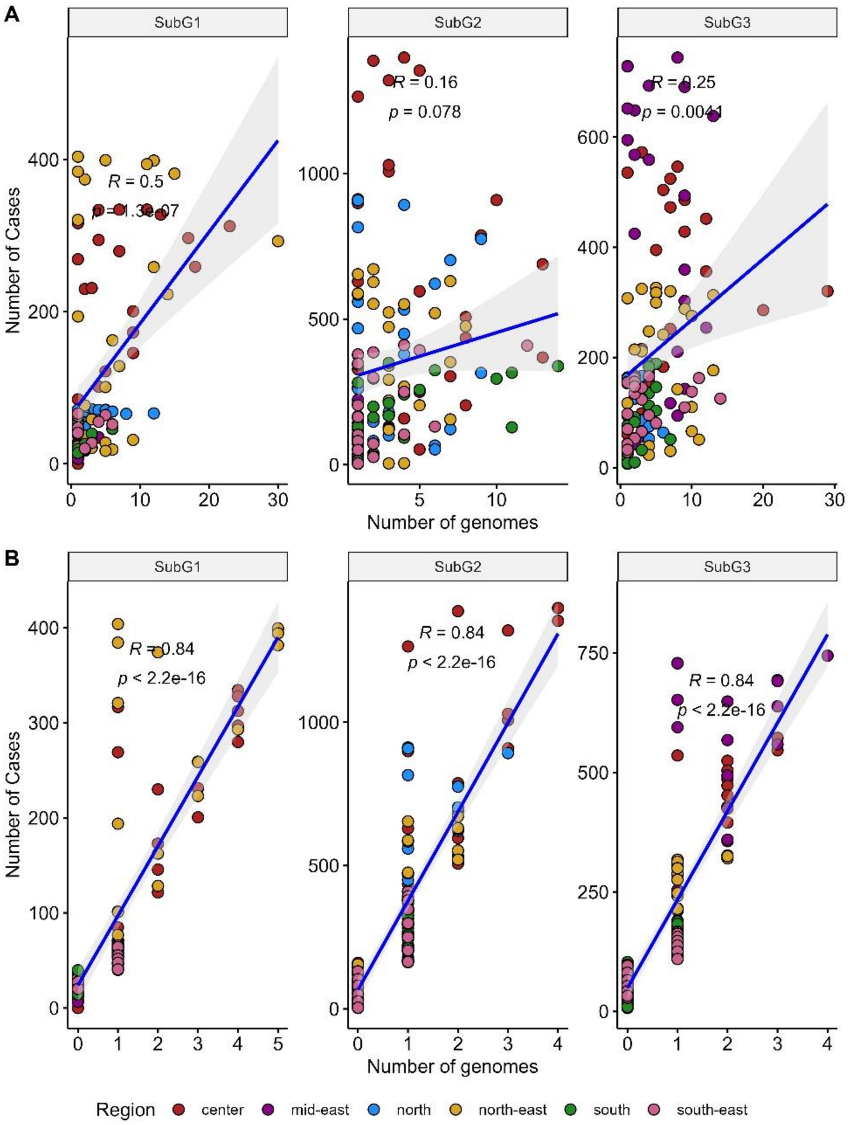

Figure S14. Correlation between number of Gamma sublineages genomes and estimated Gamma sublineages cases of the full dataset (before sampling (A)) and after the sampling (B) considering estimated Gamma sublineages cases in each Peruvian region. Each point represents the number of Gamma sublineage genomes and the estimated Gamma sublineage cases in a specific region-week combination. Points are coloured according to the region. Grey ribbons represent the 95 % confidence interval of the regression line. n = 98, 108, and118 for SubG1, SubG2, and SubG3, respectively.

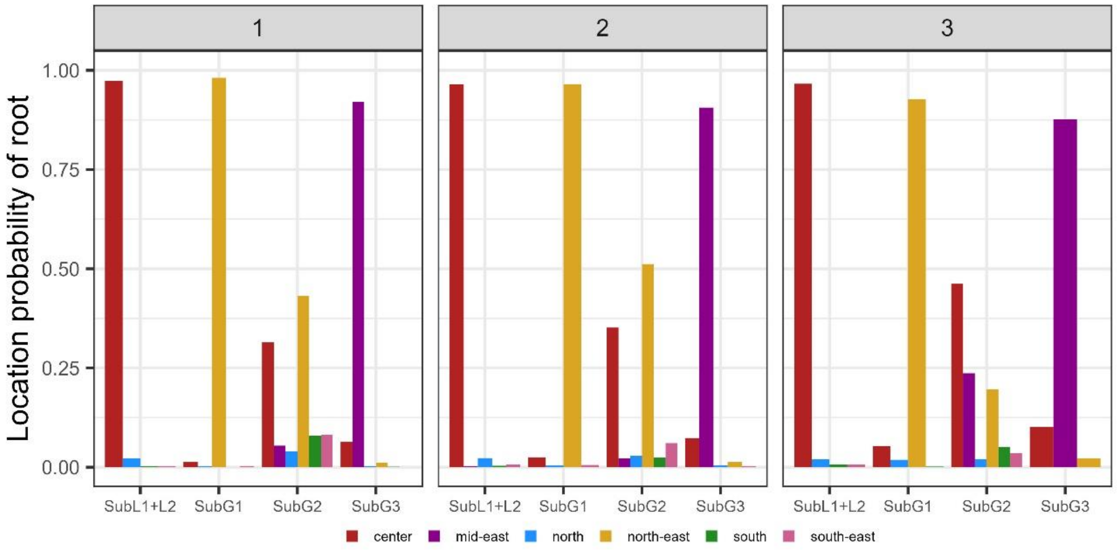

Figure S15. Estimations of location of the origin of Lambda and Gamma sublineages in different samples. Location probability of the root that represents the MRCA of the sublineage in three different samples. Bars are coloured according to the Peruvian region. The number of trees (n) from the posterior distributions that were analysed were 400 for each sample.

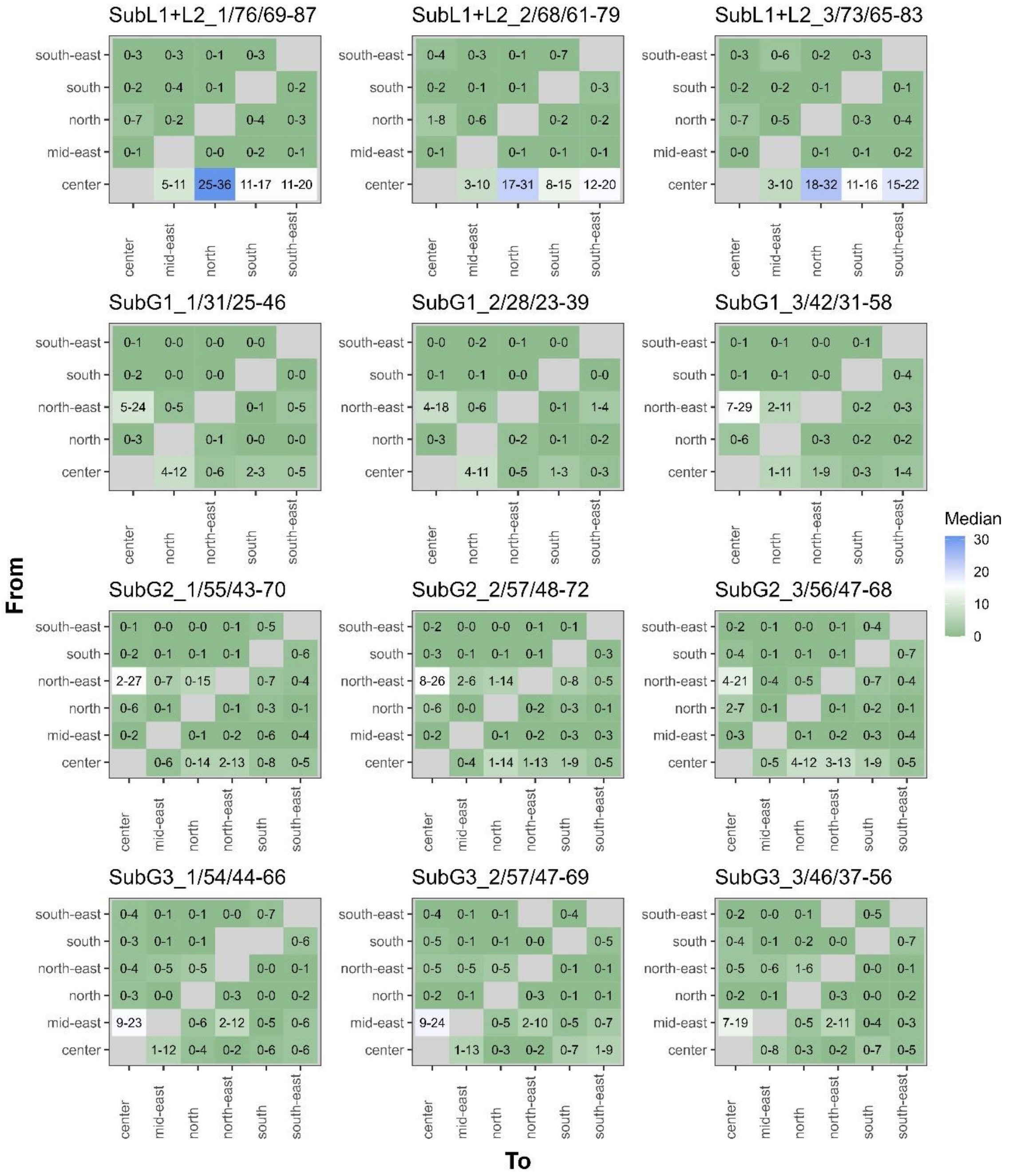

Figure S16. Number of transitions of sublineages between Peruvian regions in different samples. Matrices of the 2.5 % to 97.5 % fraction of the distribution of transitions per tree between Peruvian regions. In the y-axis is depicted the region of origin of the transition and the x-axis showed the region of destination. Squares are colored according to the median of the distribution of transitions per tree. Titles of each panel shown the name of the sublineage followe by the number of the sample separated by an underline, the median of the total transitions between regions and the overall 2.5 % to 97.5 % fraction of the distribution of transitions per tree. Each separated by "/". The number of trees (n) from the posterior distributions that were analysed were 400 for each sample.

Table S1. Classification of Peruvian cities in regions.

Region	City	Region	City
North	Tumbes	Center	Lima
	Piura		Callao
	Lambayeque	South	Ica
	Cajamarca		Arequipa
	Ancash		Moquegua
	La Libertad		Tacna
North-east	Loreto	South-east	Huancavelica
	Amazonas		Ayacucho
	San Martin		Apurimac
Mid-east	Huanuco		Cusco
	Ucayali		Puno
	Pasco		Madre de Dios
	Junin		

Table S2. Root-to-tip distance vs. sequence sampling time of each sublineage after sampling considering estimated cases in each Peruvian region.

Sublineage	R value	R^2
SubL1+SubL2	0,49	0,24
SubG1	0,60	0,36
SubG2	0,63	0,40
SubG3	0,62	0,38
SubL1	0,50	0,25
SubL2	0,52	0,27

Table S3. Distribution of quartets of each sublineage after sampling considering estimated cases in each Peruvian region.

Lineage	Quartets			
	Resolved	Partly resolved	Unresolved	Informative
SubL1+SubL2	49,47	2,97	47,56	52,44
SubG1	50,96	0,13	48,91	51,09
SubG2	43,94	0,68	55,38	44,62
SubG3	61,84	0,72	37,44	62,56
SubL1	22,28	0,75	76,97	23,03
SubL2	53,57	0,41	46,02	53,98
