# Supplementary material for: Phylodynamic of SARS-CoV-2 during the second wave of COVID-19 in Peru
Source: Nat Commun. 2023 Jun 15;14:3557. doi: 10.1038/s41467-023-39216-8 (PMC10272135; doi:10.1038/s41467-023-39216-8)
Supplement: Supplementary file 3 — Description of Additional Supplementary Files [file 41467_2023_39216_MOESM3_ESM.pdf]

File name: Supplementary Data 1
Description: Accession codes of the sequences and associated metadata used for epidemiological analysis and acknowledgements of data contributors.

File name: Supplementary Data 2
Description: Accession codes of the sequences and associated metadata used for phylodynamic analysis
