# Supplementary material for: Phylodynamic of SARS-CoV-2 during the second wave of COVID-19 in Peru
Source: Nat Commun. 2023 Jun 15;14:3557. doi: 10.1038/s41467-023-39216-8 (PMC10272135; doi:10.1038/s41467-023-39216-8)
Supplement: Supplementary file 4 — Supplementary Data 1 [file 41467_2023_39216_MOESM4_ESM.pdf]

SUPPLEMENTAL TABLE

Data Availability

GISAID Identifier:	EPI_SET_230526uh
doi:	10.55876/gis8.230526uh

All genome sequences and associated metadata in this dataset are published in GISAID's EpiCoV database. To view the contributors of each individual sequence with details such as accession number, Virus name, Collection date, Originating Lab and Submitting Lab and the list of Authors, visit 10.55876/gis8.230526uh

Data Snapshot

- EPI_SET_230526uh is composed of 9,833,385 individual genome sequences.
- The collection dates range from 2019-12-24 to 2022-04-30;
- Data were collected in 215 countries and territories;
- All sequences in this dataset are compared relative to hCoV-19/Wuhan/WIV04/2019 (WIV04), the official reference sequence employed by GISAID (EPI_ISL_402124). Learn more at https://gisaid.org/WIV04.
