# Supplementary material for: Phylodynamic of SARS-CoV-2 during the second wave of COVID-19 in Peru
Source: Nat Commun. 2023 Jun 15;14:3557. doi: 10.1038/s41467-023-39216-8 (PMC10272135; doi:10.1038/s41467-023-39216-8)
Supplement: Supplementary file 5 — Supplementary Data 2 [file 41467_2023_39216_MOESM5_ESM.pdf]

SUPPLEMENTAL TABLE

Data Availability

GISAID Identifier:	EPI_SET_230526dk
doi:	10.55876/gis8.230526dk

All genome sequences and associated metadata in this dataset are published in GISAID's EpiCoV database. To view the contributors of each individual sequence with details such as accession number, Virus name, Collection date, Originating Lab and Submitting Lab and the list of Authors, visit 10.55876/gis8.230526dk

Data Snapshot

- EPI_SET_230526dk is composed of 9,266 individual genome sequences.
- The collection dates range from 2020-07-21 to 2022-01-10;
- Data were collected in 3 countries and territories;
- All sequences in this dataset are compared relative to hCoV-19/Wuhan/WIV04/2019 (WIV04), the official reference sequence employed by GISAID (EPI_ISL_402124). Learn more at https://gisaid.org/WIV04.
