# Supplementary material for: Phylodynamic of SARS-CoV-2 during the second wave of COVID-19 in Peru
Source: Nat Commun. 2023 Jun 15;14:3557. doi: 10.1038/s41467-023-39216-8 (PMC10272135; doi:10.1038/s41467-023-39216-8)
Supplement: Supplementary file 6 — Reporting Summary [file 41467_2023_39216_MOESM6_ESM.pdf]

nature portfolio

Corresponding author(s): Santiago Justo Arevalo

Last updated by author(s): May 26, 2023

Reporting Summary

Nature Portfolio wishes to improve the reproducibility of the work that we publish. This form provides structure for consistency and transparency in reporting. For further information on Nature Portfolio policies, see our Editorial Policies and the Editorial Policy Checklist.

Statistics

For all statistical analyses, confirm that the following items are present in the figure legend, table legend, main text, or Methods section.

n/a	Confirmed	
☐	☒	The exact sample size (*n*) for each experimental group/condition, given as a discrete number and unit of measurement
☐	☒	A statement on whether measurements were taken from distinct samples or whether the same sample was measured repeatedly
☐	☒	The statistical test(s) used AND whether they are one- or two-sided<br>*Only common tests should be described solely by name; describe more complex techniques in the Methods section.*
☒	☐	A description of all covariates tested
☐	☒	A description of any assumptions or corrections, such as tests of normality and adjustment for multiple comparisons
☐	☒	A full description of the statistical parameters including central tendency (e.g. means) or other basic estimates (e.g. regression coefficient) AND variation (e.g. standard deviation) or associated estimates of uncertainty (e.g. confidence intervals)
☐	☒	For null hypothesis testing, the test statistic (e.g. *F*, *t*, *r*) with confidence intervals, effect sizes, degrees of freedom and *P* value noted<br>*Give P values as exact values whenever suitable.*
☐	☒	For Bayesian analysis, information on the choice of priors and Markov chain Monte Carlo settings
☒	☐	For hierarchical and complex designs, identification of the appropriate level for tests and full reporting of outcomes
☐	☒	Estimates of effect sizes (e.g. Cohen's *d*, Pearson's *r*), indicating how they were calculated

Our web collection on statistics for biologists contains articles on many of the points above.

Software and code

Policy information about availability of computer code

Data collection: No Software was used for data collection

Data analysis:
#databases:
Our World in Data (https://github.com/owid/covid-19-data/tree/master/public/data)
Plataforma Nacional de Datos Abiertos (https://www.datosabiertos.gob.pe/dataset/casos-positivos-por-covid-19-ministerio-de-salud-minsa)
GISAID (www.gisaid.org)
#softwares:
R v4.2.3, ViralMSA.py v1.1.30, Nextclade v2.14.1. IQ-TREE2 v2.1.2, Tempest v1.5.3, BICEPS v1.1, BEAST v2.6.7, BEAGLE v4.0.0, Tracer v1.7.2, TreeAnotator v2.7.4
#R packages:
ape v5.7-1, ggtree v3.6.2, treeio v1.22.0, dplyr v1.1.1, lubridate v1.9.2, ggplot2 v3.4.2, tidyr v1.3.0, zoo v1.8-12, ggpubr v0.6.0, ggtext v0.1.2, scales v1.2.1, ggpmisc v0.5.2, phytools v1.5-1, ggthemes v4.2.4, ggnewscale v0.4.8, reshape2 v1.4.4, openxlsx v4.2.5.2
#python packages:
biopython v1.76, pandas v1.1.3

For manuscripts utilizing custom algorithms or software that are central to the research but not yet described in published literature, software must be made available to editors and reviewers. We strongly encourage code deposition in a community repository (e.g. GitHub). See the Nature Portfolio guidelines for submitting code & software for further information.

Data

Policy information about availability of data

All manuscripts must include a data availability statement. This statement should provide the following information, where applicable:

- Accession codes, unique identifiers, or web links for publicly available datasets
- A description of any restrictions on data availability
- For clinical datasets or third party data, please ensure that the statement adheres to our policy

Publicly available datasets were analysed in this study. COVID-19 cases, deaths, stringency index and vaccinations at the country level were done based on the information of "Our World in Data" (https://github.com/owid/covid-19-data/tree/master/public/data). The number of cases by Peruvian regions were obtained from the "Plataforma Nacional de Datos Abiertos" available in https://www.datosabiertos.gob.pe/dataset/casos-positivos-por-covid-19-ministerio-de-salud-minsa. Genomic data was obtained from GISAID (https://www.gisaid.org). The accession codes of the sequences and associated metadata for epidemiology and for phylodynamic analyses used in this study are available in GISAID's EpiCoV database under EPI_SET_ID accession numbers EPI_SET_230526uh (https://doi.org/10.55876/gis8.230526uh) and EPI_SET_230526dk (https://doi.org/10.55876/gis8.230526dk), respectively . All relevant output files are available following the instructions from the GitHub repository: https://github.com/sanjusare/Phylo_SARSCOV2_Peru.

Research involving human participants, their data, or biological material

Policy information about studies with human participants or human data. See also policy information about sex, gender (identity/presentation), and sexual orientation and race, ethnicity and racism.

Reporting on sex and gender	na
Reporting on race, ethnicity, or other socially relevant groupings	na
Population characteristics	na
Recruitment	na
Ethics oversight	na

Note that full information on the approval of the study protocol must also be provided in the manuscript.

Field-specific reporting

Please select the one below that is the best fit for your research. If you are not sure, read the appropriate sections before making your selection.

☐ Life sciences ☐ Behavioural & social sciences ☒ Ecological, evolutionary & environmental sciences

For a reference copy of the document with all sections, see nature.com/documents/nr-reporting-summary-flat.pdf

Ecological, evolutionary & environmental sciences study design

All studies must disclose on these points even when the disclosure is negative.

Study description	Estimations of location and dates of the origin of the SARS-CoV-2 lineage Lambda and analysis of the migration dynamics of the Lambda and Gamma lineages in Peru
Research sample	This is a genomic epidemiology study that used available genomic data to infer origin of the Lambda variant and transmission dynamics of Gamma and Lambda inside Peru. For frequency analysis, all available genomic data was used. For origin and transmission dynamics a subsampling was done as described in methods of the main text.
Sampling strategy	Subsampling of the available genomes was done to improve correlation between estimated number of cases and the number of genomes used for phylodynamics analyses. Details of the subsampling procedures are described in methods section of the manuscript.
Data collection	This is a genomic epidemiology study that used available genomic data and associated metadata. We did not collect data.
Timing and spatial scale	Used genomes range from october 2020 to february 2022. Phylodynamic analyses were done with genomes from Argentina, Chile and Peru. Sublineages analyses were done with Peruvian genomes.
Data exclusions	Genomes with more than 290Ns and more than 10 % gaps in comparison to the reference SARS-CoV-2 genome (EPI_ISL_406801, from nt 203 to 29674) were categorized as low-quality genomes and therefore excluded from phylodynamic analyses.

Reproducibility	For all phylodynamic analyses, a minimun of four independent Bayesian analysis runs were performed, and their convergence and effective samples size were confirmed. Additionally, we take at least three subsamples to confirms the repdoucibility of the analysis.
Randomization	Not relevant as we analyzed publicly available data. SARS-CoV-2 sequences were randomly subsampled according to what we described in the methods section.
Blinding	No blinding was carried out, becuase these are not relevant for an observational study

Did the study involve field work? ☐ Yes ☒ No

Reporting for specific materials, systems and methods

We require information from authors about some types of materials, experimental systems and methods used in many studies. Here, indicate whether each material, system or method listed is relevant to your study. If you are not sure if a list item applies to your research, read the appropriate section before selecting a response.

Materials & experimental systems

n/a	Involved in the study
☒	☐ Antibodies
☒	☐ Eukaryotic cell lines
☒	☐ Palaeontology and archaeology
☒	☐ Animals and other organisms
☒	☐ Clinical data
☒	☐ Dual use research of concern
☒	☐ Plants

Methods

n/a	Involved in the study
☒	☐ ChIP-seq
☒	☐ Flow cytometry
☒	☐ MRI-based neuroimaging
